# Foreign Summary

**Published:** 1840-02-01

**Authors:** 


### FOREIGN SUMMARY.

### Velpeau's Clinical Lecture on Ophthalmia.

### No. XV.

*Non-existence of arthritic or rheumatic ophthalmia as a specific disease.*

*Arthritic Ophthalmia.*—As authors do not seem to care much about this form of specific ophthalmia, I shall only make a few brief remarks respecting it. I am myself perfectly convinced that what has been denominated arthritic ophthalmia by ophthalmologists is nothing else but iritis or choroiditis, or these two affections combined. My opinions on this subject are not formed *à priori*; they are the result of long-continued and attentive observation of disease. I have, it is true, sometimes seen the symptoms which are referred to this supposed specific form of inflammation in gouty patients, but I have much more frequently met with them in those who had never had that malady. Within the last three months, we have, as many of you well know, recognised these symptoms in at least twenty patients of all ages and of both sexes, and yet not one of them had ever been affected with gout, or even knew what the word meant. May we not also ask whether gout is a specific disease? This is a question which has not hitherto been satisfactorily answered, and yet the specific nature of gout ought certainly to be determined before we can rationally be called upon to recognise an arthritic form of ophthalmia.

The principal symptoms which are given as indicating arthritic ophthalmia are the following:—The vascularization, which is formed by vessels larger and more tortuous than in the other specific forms of inflammation, is separated from the cornea by a grayish or bluish ring or zone, about half a line in width. The sclerotica is of a deep blue or violet colour, the iris of a grayish or dirty yellow. This latter membrane appears as if it had been macerating in some fluid; and the pupil, losing its regularity of form, becomes elliptical transversely. The pain is acute, and resembles that of neuralgia.

On analyzing these symptoms, you will find that they are merely those which I described when speaking of iritis, together with some others which are referred by authors to inflammation of the choroid membrane. The increased size of the injected vessels does not depend on a gouty diathesis, but on their having become slightly varicose, and is merely a consequence of the inflammation. The blue tinge of the sclerotica shows either that the choroid membrane is inflamed, or that the sclerotica being thinner than usual, its colour is slightly modified by that membrane. This we see frequently in children, in young people of a lymphatic habit of body, and in those who are affected with hydrophthalmia. Lastly, the changes which take place in the colour and in the form of the iris, are symptoms of the inflammation of that membrane. I need not, I think, say any thing further on the subject; these considerations are quite sufficient to authorize our definitively setting aside arthritic ophthalmia.

The specific forms of ophthalmia which we have yet to examine, being those to which ophthalmologists attach the greatest importance, must be studied with care. Before, however, I enter into any details respecting the rheumatic and scrofulous forms of ophthalmia, I have a few observations to make which are of some importance.

The existence of these two specific forms of ophthalmia cannot rationally be recognised unless it be first proved that scrofula and rheumatism are really specific diseases. But, has this been done? has science come to a definitive conclusion with regard to the nature of these affections? I do not myself believe that it has; I think, indeed, that their nature is still unknown, and that their history requires revising entirely. This, however, I cannot attempt for the present, as the details into which I should be obliged to enter would lead me too far from the subject we are now discussing. I will merely observe that, in my opinion, scrofula and rheumatism only differ from other diseases by the constitution of the patient, and by the nature of the tissue which is the seat of inflammation. How, if I am correct, is it possible that they should always exercise such influence over inflammatory affections of the eye as to cause them to assume specific characters? To speak of the symptoms which are drawn from the vascularization only, how can we understand that the blood-vessels of the eye, the distribution of which is the same in every individual, should be injected in one manner in a scrofulous subject, and in another in a rheumatic subject? I must confess I cannot see how there can be any difference. I am quite willing to allow that rheumatism or scrofula, co-existing with an inflammatory affection of the eye, exercises more or less influence over that affection, as it would likewise over any other inflammatory disease; but I consider it absurd to assert, as it has been done, that a patient is rheumatic or scrofulous merely because the ophthalmia by which he is attacked presents certain characters, although he himself may offer no symptom whatever of either one or the other of these affections.

*Rheumatic Ophthalmia.*

This is one of those forms of special ophthalmia which are the most frequently alluded to by ophthalmologists. M. Sichel, in the description he gives of "rheumatic ophthalmia," says that its principal seat is the sclerotica, aponeurotic expansion of the muscles of the eye, and that portion of the conjunctiva which covers the cornea. He also says that in some instances it attacks the membrane of the aqueous humour, and the serous layer which lines the anterior surface of the iris.

In the first stage of the malady the sclerotica becomes slightly vascularized, the conjunctiva remaining healthy. The vascularization of the sclerotica is formed by numerous small vessels which run parallel to one another, are perfectly straight, of a pale carmine colour, and, converging as they approach the cornea, terminate at about the distance of a line from that membrane. At a later period the redness becomes deeper, and the injected vessels extend so as to form a zone which occupies a considerable portion of the sclerotica. At the same time, a second vascular layer becomes visible, constituted by more superficial and more voluminous vessels than the one we have just described; these vessels are moveable, follow a tortuous course, appear of a yellowish red colour, and evidently belong to the conjunctiva. These two vascular layers form round the cornea a double injected zone, the appearance of which is often followed by chemosis. The dread of light and effusion of tears are very intense. At a still more advanced period of the disease the portion of the conjunctiva which lines the external edge of the cornea becomes covered superiorly and inferiorly with small red streaks. The free margin of the eyelids is also often sympathetically affected, and assumes a bluish red tinge. If the inflammation does not abate, it sometimes extends to the corneal conjunctiva, which then becomes more or less vascularized, and presents a milky white appearance; the vessels which give rise to the vascularization seem to communicate with those of the conjunctiva. When this is the case sight is always more or less impaired, indeed sometimes it is entirely lost.

Ophthalmologists also speak of a "rheumatic keratitis," which presents, they say, the following symptoms: the cornea does not become vascularized, but its transparency is nevertheless slightly impaired. Its external surface appears uneven, and is covered with small grayish specks. At a later period effusion of a whitish or bluish matter takes place in different regions, and the haziness being much increased, vision is nearly abolished.

Such are the symptoms which are supposed to characterize rheumatic ophthalmia. If, however, we examine them attentively, we find that in reality they are merely those which I described to you when treating of keratitis. We have now several patients in our wards affected with ophthalmiæ answering perfectly to this description; yet if you will take the trouble to question them, you will not find one under the influence of a rheumatic affection. Indeed, I have repeatedly pointed out to you the fact, that the majority of both in-door and out-door patients whom we see presenting these symptoms, have never suffered from rheumatism. How can we, then, when cases of this nature are continually under our eyes, ascribe them to a specific affection, especially when we consider that simple inflammation of one or more of the membranes which enter into the formation of the eye satisfactorily accounts for their presence? It is a fact worthy of remark, that the ophthalmologists who adopt the opinions I am now opposing, do not describe a single form of inflammation, and that when they speak of traumatic inflammation they say it resembles rheumatic ophthalmia. Indeed, it is evidently not on observation but on preconceived theoretical ideas that they have founded the ideal affection we are now examining. Rheumatism attacks fibrous tissues, and as in the eye we have a fibrous tissue, the sclerotica, they have made it the seat of a rheumatic form of inflammation.

The treatment recommended against "rheumatic ophthalmia," affords additional grounds for rejecting it as a disease. The remedies which we are advised to employ are principally those which are used as special agents in the treatment of rheumatism. Now let me ask those of you who have attentively observed the cases of diseases of the eye that we have lately had in our wards, whether they have seen me resort to such measures, when I have met with the group of symptoms that is said to indicate this special affection? They will be obliged to confess, that although I have only recourse to general remedies as adjuvants, or when indicated by the general state of the patient, the plan of treatment which I have adopted has generally been followed by the most advantageous results. On the other hand, does there exist a specific agent capable of curing rheumatism? Many authors say that there are substances, such as tartarized antimony, colchicum, extract of aconitum, &c., which exercise a special influence over this disease. I have very frequently tried them, but with very slight benefit to the patient. In rheumatic affections the usual treatment of inflammatory affections seems to succeed the best.

The few remarks which I have just made will, I think, suffice to show you, that those who recognise this class of specific diseases of the eye are not only wrong in a scientific point of view, but that the ideas which they advocate have also a prejudicial effect on the treatment of ophthalmia, as they induce practitioners to resort to general measures against symptoms which would generally disappear under the influence of topical applications. I have frequently seen the inflammation subdued in a few days by the solution of the nitrate of silver, in cases which had been unsuccessfully treated by general remedies. To fully elucidate this question, it would be requisite to treat it at much greater length than I have done. I have, however, I hope, said enough to draw the attention of practitioners to the subject, and if I have succeeded in so doing I feel certain that my opinions will sooner or later predominate.

*London Medical Gazette.*

---

*State of the Kidney in Dropsy following Scarlatina.*—[*From the Proceedings of the Westminster Medical Society.*]—Mr. Streeter placed on the table the kidney of a child aged five years, who had died from anasarca succeeding to scarlet fever, with serous effusion in the cavities of the chest and abdomen, and in the substance of the lungs. The kidney was highly congested in its tubular portion. Dr. Bright had informed him that he had just had an opportunity of examining the kidney of a patient who had died under similar circumstances; the kidney was mottled, from irregular distribution of blood through its structure, with probable deposit. The first-mentioned case exhibited nothing worthy of remark, except that no medical treatment had been employed until near its termination,—the parents of the child being scarcely aware that it had scarlatina. No urine could be obtained for the purpose of being tested.

Dr. R. Willis had paid particular attention to the dangerous disease alluded to. He had originally treated it simply by purgatives and diuretics, but having examined the kidney in three fatal cases, and found unequivocal signs of inflammatory action present, he had resorted to blood-letting with the best effects; indeed, in his opinion, depletion was the only mode of checking the disease. In the cases which he had mentioned, the cortical structure, though paler than usual, was exceedingly vascular, and when cut into, a stream of blood poured out; other portions were also very vascular. He had in these cases found urea in the blood, as well as in the serum effused into the different cavities. There was urea in the serum effused in Mr. Streeter's case. He believed, in these cases, that patients died from the formation of a coagulum in the cavity of the heart. His reason for supposing this to be the cause of death was, because he had observed that, up to a certain time, the action of the heart and pulse was proper, but that suddenly the action of the two became confused, and no distinct sounds of the heart could be detected.

*London Lancet.*

